# Giant cell reparative granuloma of the hallux following enchondroma

**DOI:** 10.11604/pamj.2015.22.363.8309

**Published:** 2015-12-14

**Authors:** Khaled Kamoun, Tarak Sellami, Zied Jlailia, Layla Abid, Mourad Jenzri, Mouna Bouaziz, Omar Zouar

**Affiliations:** 1 Orthopedic Pediatric Department, Kassab Institute, Tunis, Tunisia; 2 Anatomopathology Department, Kassab Institute, Tunis,Tunisia; 3 Radiology Department, Kassab Institute, Tunis, Tunisia

**Keywords:** Tumor, bone, foot, benign

## Abstract

Giant cell reparative granuloma (GCRG) is a rare, benign intra osseous lytic lesion occurring especially in gnathis bone but also seen in feet and hands. It has similar clinical and radiological presentations than giant cell tumor, chondroblastoma, aneurysmal bone cyst, and hyperparathyroidism brown tumors but with specific histological findings We report a case of a GCRG of hallux phalanx in 18 years old patient appearing many years after enchondroma curettage and grafting. Radiographs showed a multiloculated osteolytic lesions involving whole phalanx with cortical thinning and without fluid-fluid levels in CT view. Expected to be an enchondroma recurrence, second biopsy confirmed diagnosis of GCRG with specific histological findings. Although if aetiopathogeny remains unknown, GCRG is reported to be a local non neoplasic reaction to an intraosseous hemorrhage. Our exceptional case claims that this tumor can appear in reaction to cellular disturbance primary or secondary.

## Introduction

Giant cell reparative granuloma (GCRG) is a rare, benign intraosseous lytic lesion occurring especially in gnathic bones and also in hands and feet phalanx [1–3]. It is imperative to be aware of this entity to avoid confusion with more aggressive tumors. We report a case of GCRG of the hallux appearing many years after phalanx enchondroma curettage and grafting. This case presenting an interesting hypothesis relative to the potential relationship between GCRG and phalanx enchondroma curettage and grafting. Specifically, can initial surgical treatment contributes to the progression or initiation of this type of benign tumors.

## Patient and observation

**Figure 1:**
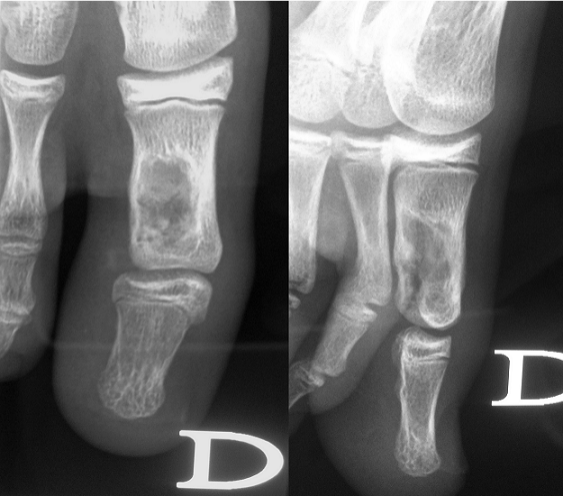
Anteroposterior and sagittal radiographs: well defined osteolytic lesion of the hallux proximal phalanx

**Figure 2:**
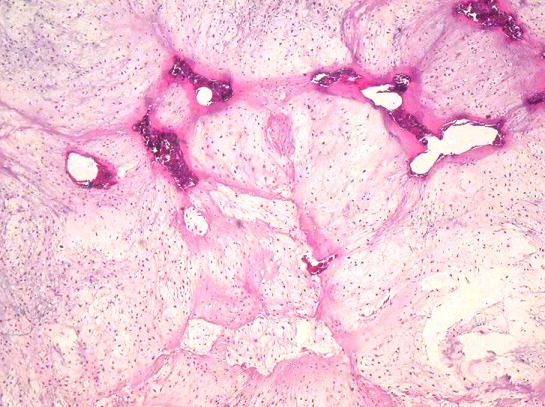
Microscopic view (Hematoxylin and Eosin stain x 250): enchondroma appearance with multiple nodules of hyaline cartilage separated from another by bone marrow

**Figure 3:**
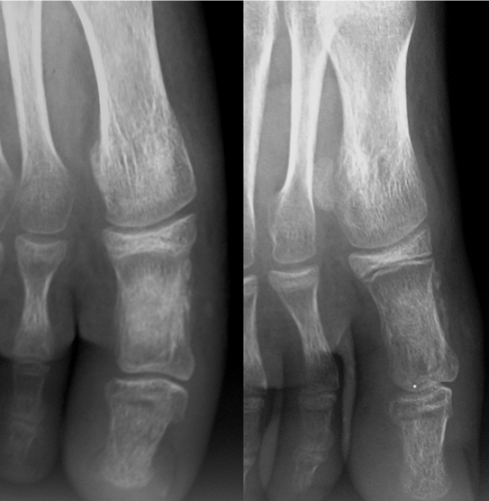
Anteroposterior and sagittal radiographs 6 mouths postoperatively: bone satisfying appearance and incorporated bone graft

**Figure 4:**
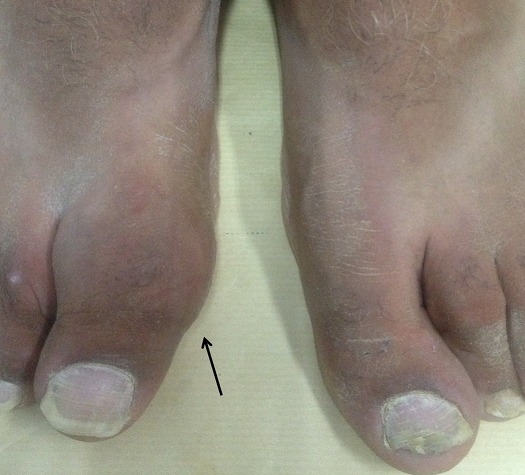
Clinical aspect of the hallux tumefaction (5 year post operatively)

**Figure 5:**
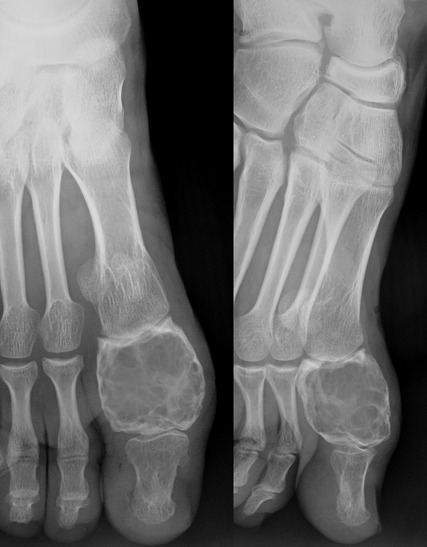
Anteroposterior and sagittal radiographs (5 year post operatively): expanding multiloculated osteolytic lesion involving the whole hallux phalanx

**Figure 6:**
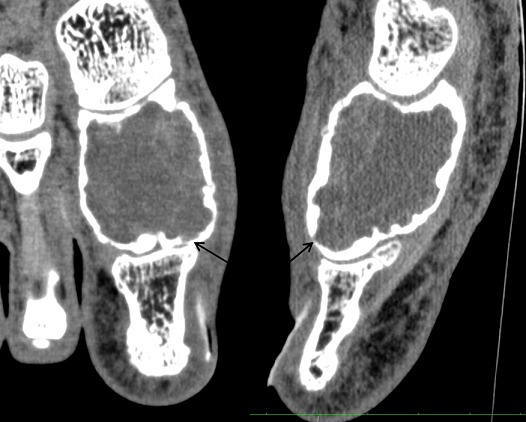
Frontal and sagittal CT view in soft tissue algorithm: expanding osteolytic lesion with focal cortical thinning and without interruption or fluid fluid levels

**Figure 7:**
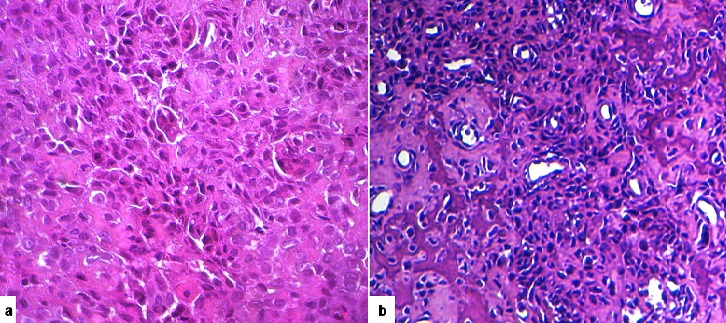
Histological GCRG appearance: a- microscopic view (Hematoxylin and Eosin stainx250): mononuclear proliferation in fibrous stroma with multiple osteoclastic- like giant cells; b- microscopic view (Hematoxylin and Eosin stain x 400). Higher power field showing extensive osteoid, numerous small capillaries and mononuclear cells loosely arranged

A 13- year-old boy presented in emergency room with left hallux painful evolving for several months without any underlying trauma. The physical examination noted a localized swallowing of the dorsum of the right hallux base with no inflammatory local signs and a pain sensitivity induced by examination. Mobility of the adjacent joint was normal and no biological abnormalities were found. Radiographic examination showed a well-defined lytic lesion of the hallux proximal phalanx. Neither cortex erosion nor soft tissues abnormalities were noticed (Figure 1). An initial biopsy was performed and showed typical features of a benign enchondroma (Figure 2). The lesion was mechanically curetted and the cavity was packed with bone graft from the isolateral iliac crest. The patients’ postoperative period was uneventful with a successful healing and a satisfying clinical and radiological evolution (Figure 3). By the fifth year postoperatively, the patient was presented with a new painful spurt of the big toe with a tumefaction that was significantly more pronounced than the initial clinical presentation (Figure 4). The radiological findings exhibited an expanding multiloculated osteolytic lesion involving whole proximal phalanx with no soft tissue nor articular involvement (Figure 5). CT scan performed confirmed the osteolytic lesion with a focal cortical thinning with no fluid-fluid levels (Figure 6). Recurrence was suspected and a new biopsy was performed and pathological analysis revealed histological aspects in favor of GCRG with multinucleated giant cells dispersed throughout hyper cellular and highly vascularized stroma, and newly formed bone and osteoid, with osteoblastic prominence (Figure 7).

## Discussion

Initially described by Jaffe [1] in 1953, the GCRG was defined as a local non neoplastic reaction to an intra osseous hemorrhage distinguishing it from other giant cell tumors. He originally described this specific entity in the gnathic bones. In 1962, Ackerman and Spjut [2] reported two cases of “giant cell lesions” occurring in the small tubular bones of the hand. The term “reparative” was added by Lorenzo and Dorfman [3] in 1980 in their additional 8 cases they reported occurring in hands and feet, noting both its inconsistent association with trauma history and its recurrence potential. GCRG is more frequent in young female patient under 30 years of age [4–6] and have similar clinical, radiological presentations [7] than giant cell tumor, chondroblastoma, aneurysmal bone cyst, and hyperparathyroidism brown tumors, but with a specific histological findings. Most GCRG seems to have an intramedullary origin and occurred on jaw (24%), small bones of the hands and feet (36%), craniofacial bone (9%), vertebrae (8%) [5] and rarely in long bones [8]. The most common clinical manifestations of GCRG include pain, swelling and palpable bone lesions and may be seen during physical exam as in our case. Radiologically, GCRG is described as an expanding osteolytic lesion with contrast enhancement that thins and expands; however, usually does not perforate the cortex of the affected bone as described in our cases [7]. Unfortunately, these radiographic features provide inadequate information to discriminate GCRG from other common bone lesions. The defining histological features were a moderately to highly cellular stroma composed of spindle to ovoid cells, multinucleated giant cells often distributed in a zonal fashion surrounding areas of hemorrhage with areas of reactive osteoid formation as seen in our case [8]. Other cases of aneurysmal bone cyst with predominantly solid areas and multinucleated giant cells had been described [9] as the “solid variant” of aneurysmal bone cyst. All these lesions appear to be related expressions of the same pathogenic mechanism with overlapping clinical, radiologic and histologic features. A series of 52 osteolytic lesions of the bones of hands and feet demonstrated difficulties to distinguish giant cell tumors from GCRG in only clinical and radiological findings [7]. Differentiation between GCRG and hyperparathyroidism brown tumor is also difficult to do with radiographic and histological features. However, by analyzing serum calcium, phosphorus,and alkaline phosphatase activity, the lesion can be diagnosed accurately [10]. Such as giant cell tumors, chondroblastomas are found in the epiphysis of a bone and expansion of the cortical bone may be present. A sclerotic border surrounding the osteolytic lesion is commonly seen in chondroblastomas. Furthermore chondroblastomas contain a mixture ofgiant cells and mononuclear cells with chondroid formation, which is not seen in giant cell reparative granuloma [7]. The aetiology of GCRG remains unknown. The multinucleated giant cells of GCRG are formed from monocyte-like and macrophage-like osteoclast precursors that differentiate into osteoclasts under the influence of osteoblast-like stromal cells. These phenomena are similar to those seen in giant cell tumors [10]. Some authors reported a concomitant enchondroma and GCRG but in different sites [8]. To our knowledge this is the first case of a secondary GCRG of hallux appearing after enchondroma surgical treatment.

## Conclusion

This case suggests a potential connection between GCRG and enchondroma and hypothesis that GCRG could appear in reaction of cellular disturbance primary or secondary to enchondroma surgical treatment.
